# Metabolomic signatures of ideal cardiovascular health in black adults

**DOI:** 10.1038/s41598-024-51920-z

**Published:** 2024-01-20

**Authors:** Shabatun J. Islam, Chang Liu, Appesh N. Mohandas, Kimberly Rooney, Aditi Nayak, Anurag Mehta, Yi-An Ko, Jeong Hwan Kim, Yan V. Sun, Sandra B. Dunbar, Tené T. Lewis, Herman A. Taylor, Karan Uppal, Dean P. Jones, Arshed A. Quyyumi, Charles D. Searles

**Affiliations:** 1 Division of Cardiology, Department of Medicine, Emory University School of Medicine, Atlanta, GA USA; 2 Department of Epidemiology, Rollins School of Public Health, Emory University, Atlanta, GA USA; 3 Department of Biostatistics and Bioinformatics, Rollins School of Public Health, Emory University, Atlanta, GA USA; 4 Department of Biomedical Informatics, Emory University School of Medicine, Atlanta, GA USA; 5 Atlanta VA Health Care System, Decatur, GA USA; 6 Nell Hodgson Woodruff School of Nursing, Emory University, Atlanta, GA USA; 7 Department of Medicine, Morehouse School of Medicine, Atlanta, GA USA; 8 Division of Pulmonary, Allergy, Critical Care and Sleep Medicine, Department of Medicine, Emory University School of Medicine, Atlanta, GA USA

**Keywords:** Epidemiology, Molecular medicine, Predictive markers, Metabolic pathways, Metabolomics

## Abstract

Plasma metabolomics profiling is an emerging methodology to identify metabolic pathways underlying cardiovascular health (CVH). The objective of this study was to define metabolomic profiles underlying CVH in a cohort of Black adults, a population that is understudied but suffers from disparate levels of CVD risk factors. The Morehouse-Emory Cardiovascular (MECA) Center for Health Equity study cohort consisted of 375 Black adults (age 53 ± 10, 39% male) without known CVD. CVH was determined by the AHA Life’s Simple 7 (LS7) score, calculated from measured blood pressure, body mass index (BMI), fasting blood glucose and total cholesterol, and self-reported physical activity, diet, and smoking. Plasma metabolites were assessed using untargeted high-resolution metabolomics profiling. A metabolome wide association study (MWAS) identified metabolites associated with LS7 score after adjusting for age and sex. Using *Mummichog* software, metabolic pathways that were significantly enriched in metabolites associated with LS7 score were identified. Metabolites representative of these pathways were compared across clinical domains of LS7 score and then developed into a metabolomics risk score for prediction of CVH. We identified novel metabolomic signatures and pathways associated with CVH in a cohort of Black adults without known CVD. Representative and highly prevalent metabolites from these pathways included glutamine, glutamate, urate, tyrosine and alanine, the concentrations of which varied with BMI, fasting glucose, and blood pressure levels. When assessed in conjunction, these metabolites were independent predictors of CVH. One SD increase in the novel metabolomics risk score was associated with a 0.88 higher LS7 score, which translates to a 10.4% lower incident CVD risk. We identified novel metabolomic signatures of ideal CVH in a cohort of Black Americans, showing that a core group of metabolites central to nitrogen balance, bioenergetics, gluconeogenesis, and nucleotide synthesis were associated with CVH in this population.

## Introduction

Cardiovascular disease (CVD) is the leading cause of death in the United States, and there are marked disparities in CVD prevalence and outcomes between Black Americans and other racial groups^1,2^. High resolution metabolomic profiling is an emerging technology that has the potential to identify pathways underlying cardiovascular health (CVH). The metabolome consists of endogenous, pharmaceutical, and chemical metabolites that fall downstream of genomic, transcriptomic, and proteomic variations^3^. Metabolomic profiling has shown great promise in elucidating novel mechanisms underlying cardiometabolic and subclinical or clinically apparent CVD^4–6^. Although Black adults suffer from a greater burden of cardiometabolic risk factors^1^, the metabolic signatures associated with CVH remain understudied in this vulnerable population^7,8^.

The purpose of this study was to determine the metabolomic signatures for CVH in Black adults without known CVD and living in the greater metropolitan area of Atlanta, GA^9^. For this study, CVH was defined by the American Heart Association’s Life’s Simple 7 (LS7) metric (score of 0 to 14 with 14 being ideal CVH), which encompasses a set of seven CVH clinical domains (four health behaviors—smoking, weight, physical activity, and diet; three health factors—blood pressure, total cholesterol, and glucose), and has been shown to predict CVD and all-cause mortality^10–13^.

## Methods

### Study design

We studied metabolomic profiles in plasma samples from participants of the Morehouse-Emory Cardiovascular (MECA) Center for Health Equity study. Platelet-free plasma samples were obtained from 375 adults living in the greater Atlanta region, aged 30 to 70, who self-identified as Black or African American. Details on study design and recruitment strategy have been described previously^9^. In brief, exclusion criteria included known CVD (e.g., myocardial infarction, congestive heart failure, cerebrovascular accident, coronary artery disease, peripheral arterial disease, atrial fibrillation, and cardiomyopathies), concomitant chronic diseases (e.g., human immunodeficiency virus, lupus, or cancer), substance abuse (alcohol or illicit drug), psychiatric illness, pregnant or lactating females, and immobility such that individuals could not increase physical activity.

Enrolled participants visited either Emory University School of Medicine or Morehouse School of Medicine where they completed a physical examination, blood draw, and questionnaires. Vital signs and anthropometric measures were recorded. All blood draws were performed after > 6 h of fasting, and cholesterol and glucose levels were measured. The presence of hypertension was verified by any of the following: current use of anti-hypertensive medications, systolic blood pressure ≥ 130 mmHg, or diastolic blood pressure ≥ 80 mmHg. The presence of diabetes mellitus was determined by either current use of diabetes medications or fasting glucose ≥ 126 mg/dL. Finally, the presence of hyperlipidemia was defined by either current use of lipid-lowering medications or fasting total cholesterol ≥ 240 mg/dL. The protocol was approved by the Institutional Review Boards at Morehouse School of Medicine and Emory University and all participants provided written informed consent. The study was conducted in accordance with the relevant guidelines and regulations of these respective institutions.

### Life’s Simple 7 score

Life’s Simple 7 (LS7) score^10^, developed by the American Heart Association, was calculated for the participants as their metric of clinical CVH. Seven clinical domains of CVH (physical activity, diet, smoking history, blood pressure, glucose, cholesterol, and BMI) were scored as 0 (poor), 1 (intermediate), or 2 (ideal), using the previously published scoring algorithm^10^. The summary score was computed by the summation of the 7 sub-scores with the range of 0 to 14, with higher scores indicating greater CVH (Table S1).

### High-resolution metabolomics (HRM)

Untargeted low molecular weight metabolic profiles (85–1250 daltons) were obtained from platelet free plasma (PFP) samples using the HRM platform described previously^14–16^. Blood was drawn into sodium citrate tubes and PFP was collected by centrifugation at 2,500 × g for 15 min followed a second centrifugation at 2,500 × g for 15 min before storage at − 80 °C. For metabolomics profiling, PFP samples were thawed and treated with acetonitrile (2:1, v/v), spiked with internal standard mix, and centrifuged at 14,000 × g for 5 min at 4 °C to remove proteins. Samples were maintained at 4 °C in an autosampler until injection and were randomized to minimize effects of instrumental drift during analysis. Three technical 10 μL aliquot replicates were analyzed on a Thermo Scientific Orbitrap Fusion Tribrid Mass Spectrometer using a Thermo Dionex Ultimate 3000 liquid chromatography system with HILIC (hydrophilic interaction liquid chromatography; ThermoFisher Scientific, Accucore, 50 × 2.1 mm, 2.6 μm) separation and electrospray ionization operated in positive mode. The flow rate of the HILIC column was maintained at 0.35 mL/min until 1.5 min, increased to 0.4 mL/min at 4 min and held for 1 min, resulting in a total analytical run time of 5 min. Mobile Phases A and B were LCMS grade water and acetonitrile, respectively. Mobile phase C was composed of 2% formic acid (v/v) in water. Mobile phase conditions consisted of 22.5% A, 75% B, 2.5% C which was held for 1.5 min, with a linear gradient to 77.5% A, 20% B, 2.5% C at 4 min, and held for 1 min. The HILIC column was then flushed for 5 min with a wash solution of 77.5% A, 20% B, 2.5% C before another injection. Mass spectral detection was completed at 120,000 resolution over a mass-to-charge ratio (*m/z*) range of 85–1275. A quality control pooled reference plasma sample (Q-Std3) was included at the beginning and end of each batch of 20 samples for quality control and quality assurance^17^. Raw data files were extracted using apLCMSv6.3.3^18^ with xMSanalyzer v2.0.7^19^, followed by batch correction with ComBat^6^. Uniquely detected ions consisting of *m/z* and retention time (RT) are referred to as metabolic features or respective metabolites, as appropriate. Metabolic feature annotation was performed using xMSannotator^20^ based on the Human Metabolome Database^21^, with only medium to high confidence (score 2 or 3) considered. Further, annotations were also conducted by matching to an in-house library of previously confirmed metabolites, allowing *m/z* difference of 10 ppm and retention time difference of 60 s, which can be considered Level 1 (highest degree of confidence in metabolite identification) per criteria of Schymanski et al.^22^.

### Metabolome wide association study (MWAS)

Prior to data analysis, feature intensities in triplicates were median summarized based on the nonzero readings, and metabolic features that had > 20% zero readings were removed. Feature intensities were log2 transformed, mean centered and scaled by standard deviation^23^. Feature intensities were regressed on the LS7 score, adjusting for age and sex or age, sex, and estimated glomerular filtration rate (eGFR)^24^. Benjamini–Hochberg false discovery rate (FDR) method was used for correction of multiple hypothesis testing. Associations were considered significant at FDR < 0.2 threshold^25^.

### Pathway enrichment analysis

After MWAS, metabolites associated with LS7 score at FDR < 0.2 were characterized for pathway enrichment using *Mummichog* *v2.0.6* software^26^. Pathways including a minimum of 3 matched metabolites were selected and annotated using the criteria described above. Five confirmed (Level 1) metabolites (glutamine, glutamate, urate, tyrosine and alanine), selected based on significant association with LS7, enrichment in pathways identified by *Mummichog*, and central to the metabolite activity network (Figure S1), were converted into concentrations as previously described^27^.

### Statistical analysis

Demographic and clinical characteristics were presented by the poor (≤ 6), intermediate (7–9) and ideal (> 10) LS7 scores for descriptive purposes only^28^. Continuous variables were reported as means (± standard deviation [SD]) and compared using ANOVA across the three categories. Categorical variables were reported as proportions (%) and compared using Chi-square tests. Concentrations of select metabolites were compared across the three scores (0 = poor health, 1 = intermediate health, 2 = ideal health) of each of the seven clinical domains of the LS7 using ANOVA. Then, using linear regression models adjusted for age and sex, concentrations of these select metabolites were compared across the three levels of each clinical domain based on the least squares means. Finally, multivariable models were built including these select metabolites adjusting for age and sex or age, sex, and eGFR, with the dependent variable being the LS7 score. Using the beta coefficient for each metabolite derived from the linear regression as weights, a metabolomics score was created summing over the weighted select metabolites. The score was standardized to a mean of 0, with a standard deviation of 1. It was then analyzed in linear regression of dependent variable LS7 score to explore the composite association of metabolites, adjusting for age and sex.

We performed all statistical analyses using R version 4.0.2 (R Foundation for Statistical Computing, Vienna, Austria). FDR corrected q value < 0.2 was considered statistically significant for the MWAS^29^. For the other analyses, *p*-values < 0.05 were considered statistically significant.

## Results

### Baseline characteristics

The mean age of the 375 of participants was 53.2 ± 10.2 years with 38.9% male. Mean LS7 score was 8.0 ± 2.2 (Table 1). Lower BMI, younger age and nonsmoking status were observed in participants with higher LS7 scores. In the overall cohort, the prevalence of hypertension was 51.2%, hyperlipidemia was 30.5%, diabetes was 20.6%, and smoking was 24.3%. The mean BMI was 32.9 kg/m^2^.

**Table 1:** Demographic and clinical characteristics of the cohort stratified by high (≥ 10), intermediate (7–9) and low (≤ 6) categories of LS7 Scores.

	Low (n = 97)	Intermediate (n = 182)	High (n = 96)	Total (n = 375)	*p* value
Demographics n(%) or mean (SD)					
Age, years	55.5 (8.1)	54.4 (9.5)	48.6 (12.0)	53.2 (10.2)	** < 0.001**
Male	36 (37.1)	65 (35.7)	45 (46.9)	146 (38.9)	0.176
Objective/Clinical Measures n(%) or mean (SD)					
Hypertension	81 (83.5)	95 (52.2)	16 (16.7)	192 (51.2)	** < 0.001**
Hyperlipidemia	45 (46.4)	60 (33.0)	9 ( 9.5)	114 (30.5)	** < 0.001**
Diabetes Mellitus	46 (47.9)	28 (15.4)	3 ( 3.1)	77 (20.6)	** < 0.001**
Current Smoker	40 (41.2)	42 (23.1)	9 ( 9.4)	91 (24.3)	** < 0.001**
Body Mass Index (BMI)	36.4 (7.5)	34.0 (8.3)	27.2 (5.7)	32.9 (8.2)	** < 0.001**
Systolic Blood Pressure (mmHg)	140.2 (19.5)	131.5 (18.4)	119.7 (15.5)	130.7 (19.4)	** < 0.001**
Diastolic Blood Pressure (mmHg)	85.1 (12.2)	81.4 (11.0)	73.3 (9.4)	80.3 (11.8)	** < 0.001**
Total Cholesterol (mg/dL)	200.9 (43.9)	196.8 (37.9)	172.9 (30.4)	191.7 (39.4)	** < 0.001**
HDL (mg/dL)	54.1 (16.8)	56.49 (16.6)	61.5 (17.9)	57.1 (17.1)	**0.008**
LDL (mg/dL)	121.7 (38.1)	119.5 (34.5)	95.3 (25.2)	113.8 (35.0)	** < 0.001**
Triglycerides (mg/dL)	129.0 (83.4)	104.9 (46.9)	75.5 (37.7)	103.6 (59.9)	** < 0.001**
Fasting Glucose (mg/dL)	121.4 (43.7)	98.9 (31.9)	90.0 (23.8)	102.4 (35.6)	** < 0.001**
Total LS7 score	5.2 (0.82)	7.9 (0.82)	10.8 (0.92)	7.97 (2.20)	** < 0.001**

### Metabolome-wide association study (MWAS)

**Figure 1:**
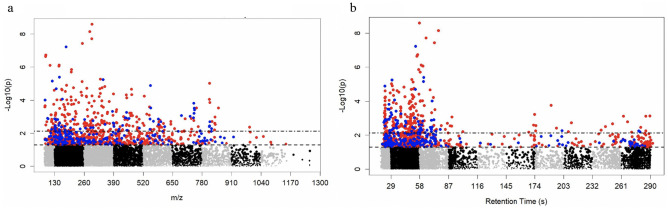
Manhattan plot (− log *p* vs (**a**) m/z or (**b**) retention time) of metabolites determined by MWAS to be associated with LS7 (as a continuous variable) after adjusting for age and sex. 8211 metabolic features underwent MWAS analysis. 301 metabolic features were differentially expressed at FDR < 0.2; 232 metabolic features colored red were lower with higher LS7 and 69 metabolic features colored blue were higher with higher LS7. Retention time is expressed in seconds.

Untargeted, high resolution plasma metabolomics profiling of 375 MECA participants was performed using liquid chromatography/mass spectrometry. A total of 14,501 metabolic features were detected; metabolic features that had > 20% zero readings were removed and 8,211 underwent further analysis. After adjusting for age and sex, MWAS identified 301 metabolic features that were associated with the LS7 score at FDR cutoff < 0.2; 232 metabolic features were lower and 69 metabolic features were higher with higher LS7 (Fig. 1, Table 2, Table S2).

**Table 2:** Select metabolites from the metabolome wide association study (MWAS) that were significantly associated with ideal cardiovascular health as defined by AHA Life’s Simple 7 (LS7). For list of other metabolites, please refer to Table S2.

Name	m/z_RT(sec)	Beta	Standard Error	*p* value	FDR Q value	HMDB ID
Lower expression with higher LS7						
5-Deoxyadenosine	mz252.1078_t72.7	− 0.131	0.023	3.68E-08	7.56E-05	HMDB00101
Alanine	mz90.055_t55.2	− 0.124	0.024	2.22E-07	0.00026	HMDB00056
Glutamate	mz148.0605_t61.2	− 0.117	0.023	8.51E-07	0.00070	HMDB00148
Urate	mz169.0359_t50.8	− 0.087	0.023	0.00018	0.023	HMDB00289
N-amidino-L-aspartate	mz176.0659_t38.7	− 0.086	0.024	0.00037	0.039	HMDB03157
Ferulate	mz195.0655_t59.4	− 0.082	0.024	0.00072	0.061	HMDB00954
Leucine/Isoleucine	mz132.1019_t39.1	− 0.078	0.023	0.00086	0.064	HMDB00172
Indole-3-acetate	mz176.0705_t24.9	− 0.076	0.024	0.0017	0.097	HMDB00197
Proline	mz116.0707_t60.4	− 0.075	0.024	0.0020	0.102	HMDB00162
Tyrosine	mz182.0811_t49.6	− 0.073	0.024	0.0027	0.119	HMDB00158
3-Hydroxybenzaldehyde	mz123.0441_t49.6	− 0.073	0.024	0.0026	0.119	HMDB01870
Phenylpuruvate	mz165.0546_t49.3	− 0.071	0.024	0.0036	0.138	HMDB00205
1-Methyladenosine	mz282.1189_t54.1	− 0.066	0.024	0.0060	0.183	HMDB03331
Higher expression with higher LS7						
Maleamate	mz116.0343_t74.3	0.067	0.024	0.0057	0.179	NA
Oxoproline	mz130.05_t65.3	0.068	0.024	0.0050	0.165	HMDB00267
Glutamine	mz147.0766_t70.5	0.071	0.024	0.0033	0.133	HMDB00267
Homocysteine	mz136.0427_t53.5	0.102	0.024	2.00E-05	0.0064	HMDB00742
Methyladenine	mz150.0766_t62.1	0.110	0.023	4.08E-06	0.0026	HMDB02099

HMDB ID- Human Metabolome Database Identification; m/z- mass-to-charge ratio; RT- Retention time, expressed in seconds; NA- Not Annotated in HMDB;

### Metabolic pathways analysis

**Figure 2:**
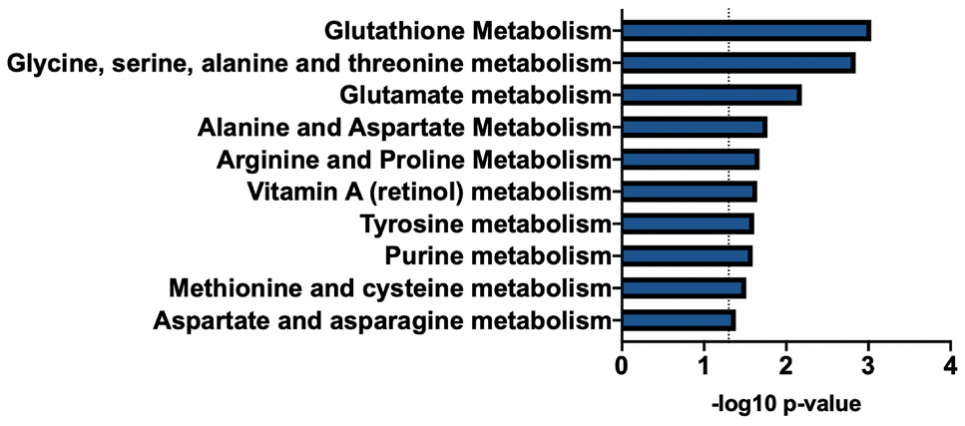
Metabolic pathways enriched for metabolites associated with LS7. Pathway analysis was performed using *Mummichog* software (version 2.0.6). The 301 metabolic features associated with LS7 by MWAS were entered into the analysis. Significantly enriched pathways (*p* < 0.05) are shown.

Using *Mummichog*, pathway enrichment analysis of the 301 metabolic features associated with LS7 identified 10 metabolic pathways (*p*-value < 0.05), including those involved in metabolism of glutathione, several amino acids, vitamin A, and purine (Fig. 2). Several metabolites identified by MWAS and listed in Table 2 matched to multiple pathways (Table S3). For example, alanine, glutamine, and glutamate matched to the glutathione, glutamate, alanine and aspartate, and tyrosine metabolism pathways. Visualization of the metabolite activity network, which combined analyses of identified pathways with metabolite modules showed that the amino acids glutamate, glutamine, alanine, and tyrosine were central nodes connecting different metabolic pathways (Figure S1).

### Association of metabolite concentrations with clinical domains of LS7

**Figure 3:**
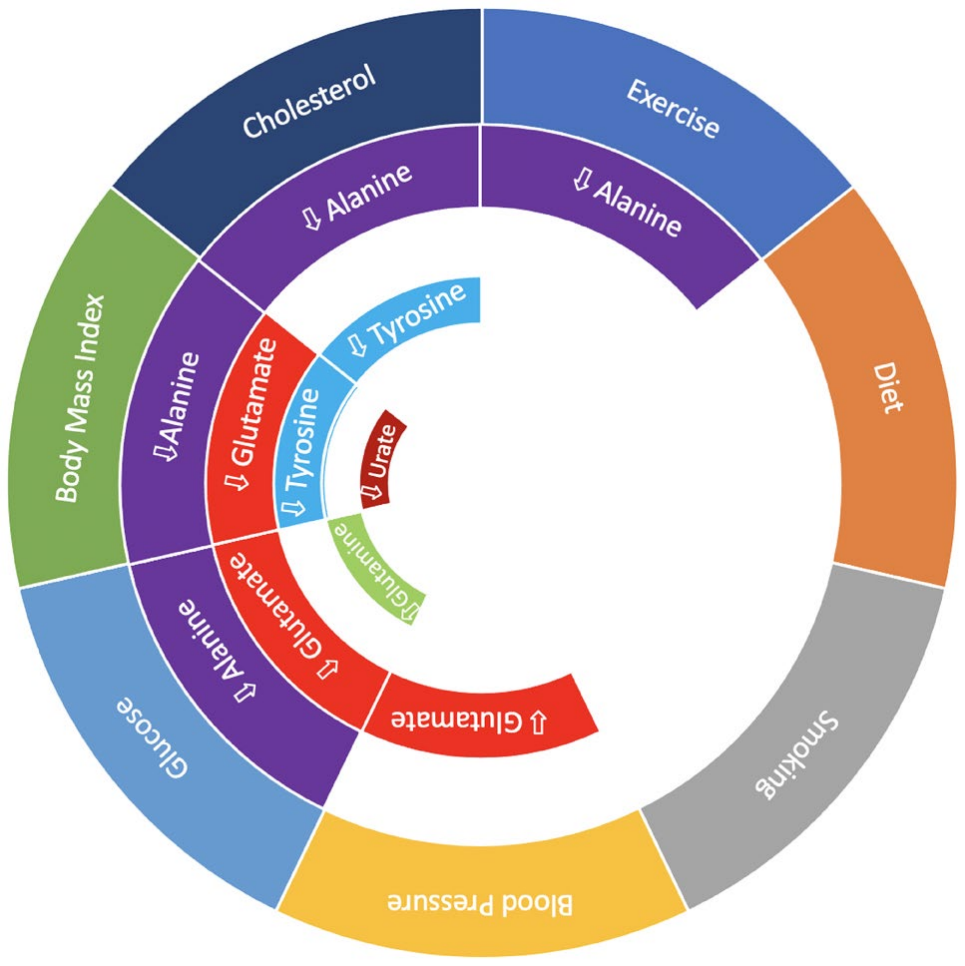
LS7 clinical domains associated with metabolite concentrations. Concentrations were compared across three scores (0, 1, 2) of the seven clinical domains of the LS7 using ANOVA. Upward arrows indicate that metabolite concentration increased with respective increase in clinical domain score, while downward arrows indicate metabolite concentration decreased with respective increase in LS7 score.

To further examine the relationship between plasma metabolite levels and CVH, we compared concentrations of five confirmed (Level 1) metabolites (alanine, glutamine, glutamate, tyrosine, and urate) across clinical domains of LS7. All five metabolites were identified by MWAS to be associated with LS7 score when adjusted for age and sex (Table 2) or adjusted for age, sex, and eGFR (Table S5), were enriched in the metabolic pathways identified by *Mummichog* (Fig. 2, Table S3), and were central nodes of the metabolite activity network (Figure S1). Furthermore, the levels of these metabolites were stable across the samples as indicated by relatively low coefficients of variation (CV)—glutamine, 1.24%; glutamate—3.06%; Urate—3.39%; tyrosine—1.58%; alanine—1.89%. Metabolite concentrations were determined as described by Liu et al.^27^ When we compared metabolite concentrations across the three scores (0 = poor health, 1 = intermediate health, or 2 ideal health) for each clinical domain of the LS7, we found that glutamate concentrations were higher with poor blood pressure, glucose and BMI scores, while glutamine concentrations were lower with poor glucose score. Urate concentrations were higher with poor BMI score, and tyrosine concentrations were higher with poor cholesterol and BMI scores. Alanine concentrations were higher with poor cholesterol, glucose, and BMI scores and lower with ideal physical activity (Fig. 3, Table 3). Analysis of concentrations across LS7 clinical domains adjusted for age and sex demonstrated showed similar results (Table S4). These data suggest that plasma metabolite levels were influenced by individual clinical domains of LS7, indicating that the expression profile of these five metabolites can reflect individual CVH.

**Table 3:** Concentrations in μM of select metabolites compared across LS7 clinical domains (unadjusted).

		Glutamine	Glutamate	Urate	Tyrosine	Alanine
Blood Pressure	Poor	412 ± 121	35 ± 19	154 ± 77	71 ± 22	269 ± 81
	Intermediate	406 ± 146	29 ± 14	155 ± 73	69 ± 23	261 ± 89
	Ideal	417 ± 140	28 ± 13	142 ± 60	70 ± 22	246 ± 64
	*p* value	0.87	**0.001**	0.37	0.77	0.09
Cholesterol	Poor	431 ± 102	34 ± 14	155 ± 72	79 ± 21	279 ± 97
	Intermediate	399 ± 143	33 ± 19	153 ± 82	68 ± 23	270 ± 85
	Ideal	417 ± 129	30 ± 15	149 ± 64	70 ± 22	250 ± 70
	*p* value	0.27	0.13	0.88	**0.03**	**0.02**
Glucose	Poor	373 ± 137	37 ± 20	167 ± 91	68 ± 22	301 ± 110
	Intermediate	417 ± 127	36 ± 17	157 ± 74	73 ± 23	277 ± 70
	Ideal	419 ±132	29 ± 15	146 ± 66	70 ± 23	247 ± 70
	*p* value	**0.045**	** < 0.001**	0.10	0.39	** < 0.001**
Body Mass Index (BMI)	Poor	410 ± 131	33 ± 16	159 ± 75	74 ± 23	276 ± 85
	Intermediate	415 ± 129	29 ± 15	143 ± 63	65 ± 19	248 ± 71
	Ideal	411 ± 146	29 ± 21	136 ± 72	65 ± 21	228 ± 64
	*p* value	0.96	**0.05**	**0.038**	**0.001**	** < 0.001**
Exercise	Poor	386 ± 136	36 ± 15	158 ± 88	70 ± 22	266 ± 57
	Intermediate	415 ± 123	33 ± 19	146 ± 71	72 ± 22	279 ± 89
	Ideal	412 ± 138	30 ± 16	154 ± 71	70 ± 23	250 ± 76
	*p* value	0.54	0.068	0.52	0.75	**0.005**
Diet	Poor	424 ± 126	29 ± 14	154 ± 71	72 ± 20	271 ± 83
	Intermediate	405 ± 132	32 ± 18	149 ± 74	69 ± 23	256 ± 80
	Ideal	405 ± 168	36 ± 19	161 ± 61	76 ± 26	262 ± 71
	*p* value	0.45	0.12	0.68	0.19	0.26
Smoking	Poor	409 ± 129	33 ± 20	156 ± 81	745 ± 24	255 ± 80
	Intermediate	341 ± 141	33 ± 13	158 ± 89	71 ± 28	277 ± 71
	Ideal	416 ± 133	31 ± 16	150 ± 68	69 ± 21	263 ± 81
	*p* value	0.10	0.41	0.75	0.09	0.55

### Metabolite risk score and cardiovascular health in black adults

**Figure 4:**
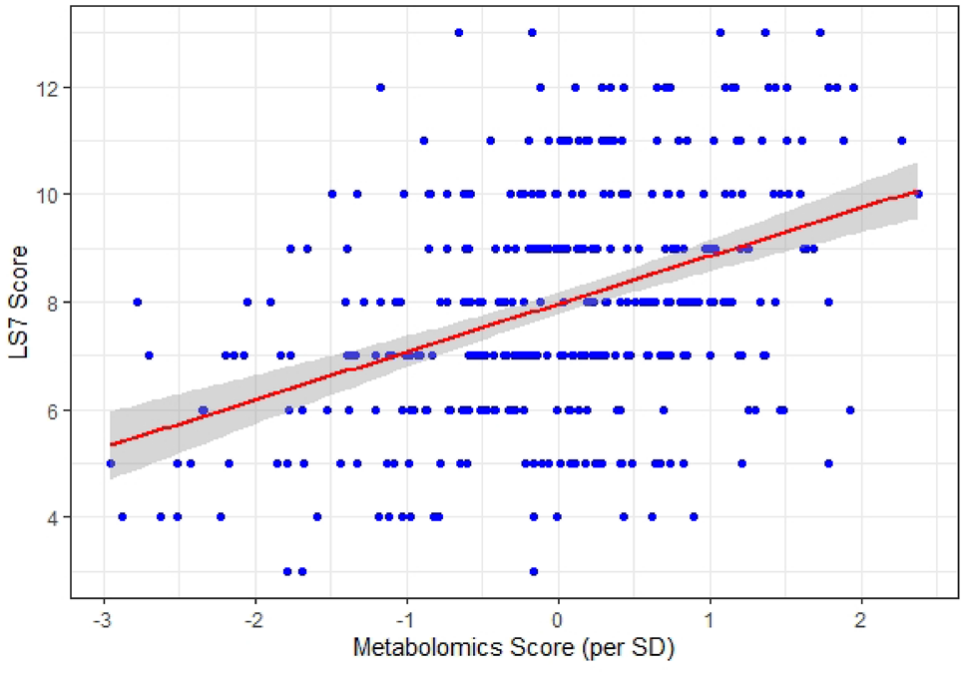
Relationship between metabolite risk score and LS7. One standard deviation increase in the score corresponded to 0.88 higher LS7 score.

A multivariable model using the concentrations of the five metabolites demonstrated that all five were associated with LS7 score, independent of each other (Tables 4, S7). After adjusting for age and sex, the model demonstrated that one standard deviation increase in the respective metabolite concentration was associated with a 0.45 unit higher LS7 score for glutamine, and 0.28 to 0.54 unit lower LS7 scores for the other metabolites (Table 4). Similar results were observed for a multivariable model that adjusted for age, sex, and eGFR (Table S7). The weighted metabolite risk score comprised of these five metabolites continued to be associated with total LS7 score, where one standard deviation increase in the score corresponded to a 0.88 unit higher LS7 score (*p* < 0.001, Fig. 4), which translates to a 10.4% lower incidence of lifetime CVD^30^. A similar relationship between metabolite risk score and LS7 score was observed when the metabolite risk score was adjusted for age, sex, and eGFR—estimate 0.88 unit higher LS7 score for one standard deviation increase in metabolite risk score (95% CI 0.69, 1.08; p = 5.69E-17). These findings emphasize the importance of non-essential amino acid and purine metabolism in CVH and demonstrate the ability of plasma concentrations of glutamate, glutamine, alanine, tyrosine, and urate to predict CVH.

**Table 4:** Multivariable linear regression demonstrated that in a model adjusting for age and sex, all five of the metabolites were independently associated with LS7.

Name	m/z_RT(sec)	Beta*	Standard Error	*p* value
Glutamine	mz147.0766_t70.5	0.45	0.12	< 0.001
Glutamate	mz148.0605_t61.2	− 0.32	0.11	< 0.001
Urate	mz169.0359_t50.8	− 0.38	0.11	< 0.001
Tyrosine	mz182.0811_t49.6	− 0.28	0.12	0.02
Alanine	mz90.055_t55.2	− 0.54	0.12	< 0.001

*Beta: increase in LS7 per one standard deviation increase in respective metabolite concentration.

## Discussion

**Figure 5:**
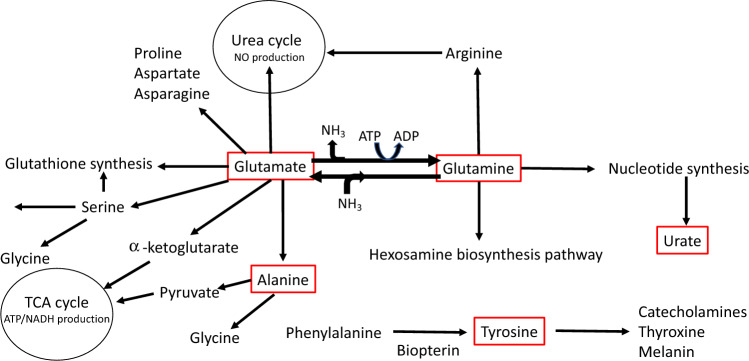
Metabolites predictive of CVH in MECA are central to essential metabolic pathways. Five metabolites (red rectangles) that were associated with CVH and were the focus of current study are central components of key metabolic processes.

Herein, we used high resolution metabolomics profiling, advanced data extraction algorithms, and pathways analysis to examine metabolites and pathways underlying CVH in a cohort of Black participants without known CVD and living in the greater metro area of Atlanta, GA. CVH was assessed by AHA LS7 score, which incorporates key clinical risk factors for CVD and has been previously demonstrated to be a surrogate for CVH^30^. We identified novel metabolomic signatures that included five key metabolites (glutamine, glutamate, urate, tyrosine, and alanine) linked to energy production, nitrogen balance, gluconeogenesis, and the metabolic syndrome (Fig. 5)^5,31–40^. Subsequently, we created a novel metabolomics risk score to predict CVH in Black adults.

We found that the concentrations of these five metabolites in the plasma were specifically associated with poor, intermediate, or ideal scores in the cardiometabolic domains of the LS7 score. Prior studies have demonstrated similar association between levels of non-essential amino acids (e.g. glutamate, glutamine, alanine, etc.) and cardiometabolic risk or disease, such as diabetes, in multiethnic populations not enriched for Black adults^8,31–34^. Cheng et al. corroborated a metabolomic signature involving non-essential amino acids, such as alanine, with metabolic syndrome within a mainly white cohort enrolled in the Framingham Heart Study^31^. They also demonstrated an association of high glutamine to glutamate ratio with lower risk of incident diabetes. Similar associations between non-essential amino acid levels and cardiometabolic risk were noted in Asian^32,33^ and Mediterranean^34^ cohorts. To our knowledge, only two other studies, both conducted within the Jackson Heart Study cohort, have assessed metabolomic signatures in Black adults but in the context of incident coronary heart disease^7^ and heart failure^8^. Thus, the current study is the first to demonstrate that CVH (or CV risk factors) in Black Americans is associated with metabolomic signatures similar to those reported for other racial/ethnic cohorts. In particular, the current study demonstrated the importance of glutamate and glutamine metabolism in this cohort. In addition, other novel metabolic pathways associated with CVH were identified. Whether these other pathways are only relevant to CVH in Black adults require further study in multi-ethnic cohorts.

We found that concentrations of both glutamine and glutamate varied with glucose levels, while glutamate concentrations also correlated with blood pressure and BMI. Glutamine is one of the most abundant amino acids in the body, classified as a conditionally essential amino acid, and has a critical role in nitrogen balance, providing intermediates to the tricarboxylic acid (TCA) cycle (anapleurosis), immunity, and pH homestasis^5,35–40^. Glutamine is mainly synthesized from glutamate and ammonia by the enzyme glutamine synthetase (Fig. 5)^7^. The pathophysiology of cardiometabolic disease is complex and disturbances in glutamine/glutamate metabolism have been implicated in the development of metabolic risk in multiethnic communities^5,31–34^. The role of glutamine/glutamate in CVH is likely mediated through multiple mechanisms. First, glutamine has potent antioxidant, anti-inflammatory, and anti-apoptotic effects by stimulating glutathione, heat shock proteins, and heme oxygenase-1^5,41^. Glutamine also stimulates nitric oxide bioavailability by increasing arginine synthesis that may maintain normal blood pressure^42^. We note here that in the current study glutamate levels were higher with higher blood pressure.

Glutamine can improve glucose homeostasis by stimulating release of glucagon-like peptide-1, externalization of glucose transporters, insulin release by pancreatic β-cells, transcription of insulin-dependent genes, and increased insulin disposition^43^. Glutamine also increases the transamination of pyruvate to alanine, which is directly involved with the TCA cycle and is involved in modulating obesity by being a strong promoter of gluconeogenesis^44^. Dysregulation in glutamate/glutamine metabolism can lead to higher levels of alanine^45^, which has been shown to be associated with increased Type 2 diabetes risk^46^. In our study, we noted that lower levels of alanine were associated with ideal glucose, BMI, cholesterol scores, as well as the modifiable component of physical activity. Based on these data, we hypothesize that the well-recognized beneficial effects of physical activity on CVH are mediated through changes in the metabolome, including changes in alanine metabolism, but future studies are required to determine whether health interventions that involve increased physical activity can modify alanine levels.

Tyrosine was another one of the select metabolites that was associated with BMI, as well as the cholesterol subcomponent of LS7 score. Previously, elevated levels of tyrosine have shown positive associations with insulin resistance and Type 2 diabetes in multiple non-Black cohorts^31,47–50^. Catecholamines (dopamine, norepinephrine, epinephrine) are neurotransmitters synthesized from tyrosine^51,52^. We speculate that changes in tyrosine can likely lead to changes in physiological and behavioral functions that can impact CVH in Black adults but future studies are required.

Our final metabolite of interest was urate or uric acid, the concentration of which was associated with BMI. Urate has long been established as associated with cardiovascular disease^53^. Urate is a major product of purine metabolism and has been found to impair nitric oxide synthesis and promote endothelial dysfunction, which is pro-thrombotic, pro-inflammatory, pro-vasoconstrictive, and increases risk of incident CVD^54,55^. While we did not find an association between urate levels and risk factors directly linked to endothelial dysfunction such as hypertension, we did note, in accordance with prior studies, that high urate levels were associated with obesity and as such, remain an important marker for CVH^56^.

As shown in Fig. 5, the five metabolites (glutamine, glutamate, alanine, tyrosine, and urate) that were the focus of this study are known to be central elements to key metabolic processes. Each of them has been previously shown to be associated with CVH. While Pearson correlation analysis revealed associations between expression of each metabolite and expression of other metabolites (Table S6), we uniquely demonstrate that when assessed together, these metabolites were independent predictors of CVH in Black adults, and the novel metabolomics risk score derived from the concentrations of these metabolites was similarly predictive. Prior risk scores have been developed from metabolomic signatures to predict incident coronary heart disease^29,57,58^, but, to our knowledge, we are the first to incorporate the concentration of these metabolites into a risk prediction tool for CVH.

While major limitations of this study are that it is single center, and it examined a relatively small cohort, we are among the first to demonstrate the molecular basis of CVH *within* Black Americans, a population underrepresented in scientific literature but suffers from disparate levels of CVD. Other limitations of the study are the need for replication in a separate cohort, and, given the cross-sectional design, the inability to address causality, which would require longer term follow-up. This study used the LS7 score as a surrogate for CVH, so longer-term follow-up of participants’ outcomes would also be needed to determine whether the five highlighted metabolites can predict CVH better than clinical risk factors. Furthermore, while we explored several metabolic pathways that were associated with CVH, we did not further explore others that were identified in our analysis, including pathways responsible for retinol and arginine metabolism, which require consideration in future studies. Lastly, while the concentrations of the metabolites examined in this study varied with cardiometabolic risk factors, none of them varied with diet or smoking, factors known to be important determinants of CVH. Future study involving a more detailed assessment of diet will be needed to determine its influence on the metabolomic signature associated with CVH in Black adults. Future studies will also need to examine the interaction of social and environmental factors with metabolomic signatures. Despite these limitations, we report several novel findings and present a new metabolomics risk score, which demonstrated that CVH was associated with concentrations of five metabolites, pointing to the importance of non-essential amino acid and purine metabolism in CVH and the potential of the five highlighted metabolites as therapeutic targets. Additional studies are required to validate this score in multiethnic populations and prospectively follow participants for development of cardiovascular disease.

### Supplementary Information

Supplementary Information 1.

Supplementary Table S2.

## Data Availability

The datasets generated during and/or analyzed during the current study are not publicly available due to sensitive nature of personalized healthcare data but are available from the corresponding author on reasonable request.

## Abbreviations

BMI: Body Mass Index
CI: Confidence interval
CV: Cardiovascular
CVD: Cardiovascular disease
CVH: Cardiovascular health
MECA: Morehouse-Emory Cardiovascular Center for Health Equity
LS7: Life’s Simple 7
SD: Standard deviation
