# Supplementary material for: Metabolomic signatures of ideal cardiovascular health in black adults
Source: Sci Rep. 2024 Jan 20;14:1794. doi: 10.1038/s41598-024-51920-z (PMC10799852; doi:10.1038/s41598-024-51920-z)
Supplement: Supplementary file 1 — Supplementary Information 1. [file 41598_2024_51920_MOESM1_ESM.docx]

**Table S1. American Heart Association Life’s Simple 7 (LS7) scoring algorithm***

|  | **Score categories of LS7 domains** | | |
| --- | --- | --- | --- |
|  | **Poor (0)** | **Intermediate (1)** | **Ideal (2)** |
| **Smoking** | Currently smoking | Former smoker, quit ≤ 12 months ago | Never smoker or quit >12 months ago |
| **Body mass index** | ≥ 30.0 kg/m^2^ | 25.0 - 29.9 kg/m^2^ | <25.0 kg/m^2^ |
| **Physical activity** | None | 1 - 149 min/week moderate intensity, 1 - 74 min/week vigorous intensity, or 1 - 149 min/week moderate & vigorous intensity | ≥ 150 min/week moderate intensity, ≥ 75 min/week vigorous intensity, or ≥ 150 min/week moderate & vigorous intensity |
| **Healthy Diet Score**^†^ | 0 – 1 components | 2 – 3 components | 4 – 5 components |
| **Cholesterol** | ≥ 240 mg/dL | 200 - 239 mg/dL (untreated) or treated to goal | <200 mg/dL (untreated) |
| **Blood pressure** | SBP ≥ 140mmHg or DBP ≥ 90mmHg | SBP 120 – 139mmHg, DBP 80 – 89mmHg, or treated to goal | SBP < 140mmHg and DBP < 90mmHg |
| **Fasting glucose** | ≥ 126 mg/dL | 100 - 125 mg/dL (untreated) or treated to goal | <100 mg/dL (untreated) |

* Adopted from the report from the Goals and Metrics Committee of the Strategic Planning Task Force of the American Heart Association.^9^

^†^ Healthy diet score is assessed based on the 5 aspects of diet selected by the American Heart Association: fruits/vegetables (≥ 4.5 cups/day), fish (≥two 3.5-oz servings/week), fiber-rich whole grains (≥ 1.1g of fiber per 10g of carbohydrates, sodium (<1,500mg/day), and sugar-sweetened beverage (<450kcal [36oz]/week)

Abbreviations: LS7 = Life’s Simple 7, SBP = systolic blood pressure, DBP = diastolic blood pressure

**Table S2:** Metabolites from the metabolome wide association study (MWAS) that were significantly associated with ideal cardiovascular health as defined by AHA Life’s Simple 7 (LS7).

**Please see attached file**

*Abbreviations:* HMDB ID- Human Metabolome Database Identification; m/z- mass-to-charge ratio; RT- Retention time in seconds; NA- Not Annotated in HMDB;

**Table S3:** Metabolites that matched pathways from the *Mummichog* analysis. The annotation of alanine, glutamate, glutamine, urate, proline, oxoproline were Level 1 confirmed (highest degree of confidence in metabolite identification) per criteria of Schymanski et al.^19^

| **Pathways** | **Overall Size** | **Pathway Size** | **p-value** | **Overlap Empirical Compounds** |
| --- | --- | --- | --- | --- |
| Glutathione Metabolism | 3 | 11 | 0.00092 | **Alanine, Oxoproline, Glutamate** |
| Glycine, serine, alanine and threonine metabolism | 6 | 44 | 0.00143 | 2-Aminoacrylate, 3-Phospho-D-glycerate, isobutyrylglycine, **Glutamate,** Betaine aldehyde, **Alanine** |
| Glutamate metabolism | 3 | 10 | 0.00647 | **Alanine, Glutamine, Glutamate** |
| Alanine and Aspartate Metabolism | 3 | 13 | 0.01689 | **Alanine, Glutamine, Glutamate** |
| Arginine and Proline Metabolism | 4 | 34 | 0.02109 | **Oxoproline, Glutamine, Glutamate, Proline** |
| Vitamin A (retinol) metabolism | 3 | 15 | 0.02252 | anhydroretinol, 9-cis-retinol, UDP-D-glucuronate |
| Tyrosine metabolism | 9 | 82 | 0.02454 | 2-Aminoacrylate, **Glutamine,** 3,4-Dihydroxymandelate, N-Acetylarylamine,4-Coumarate, **Alanine, Glutamate,** UDP-D-glucuronate, N-acetyl-L-alanine |
| Purine metabolism | 5 | 36 | 0.02563 | **Urate, Glutamine,** Xanthine, **Glutamate,** 5'-Deoxyadenosine |
| Methionine and cysteine metabolism | 4 | 49 | 0.03059 | Thiosulfate, **Glutamate**, 2-Aminoacrylate, Adenosine 5'-phosphosulfate |
| Aspartate and asparagine metabolism | 5 | 52 | 0.04092 | **Glutamine, Glutamate, Proline, Oxoproline,** N2-Acetyl-L-ornithine |

**Table S4:** Concentrations in μM of select metabolites compared across LS7 clinical domains adjusted for age and sex.

|  |  | **Glutamine** | **Glutamate** | **Urate** | **Tyrosine** | **Alanine** |
| --- | --- | --- | --- | --- | --- | --- |
| **Blood Pressure** | **Poor** | 406 ± 20 | 34 ± 2 | 155 ± 11 | 71 ± 3 | 267 ± 12 |
|  | **Intermediate** | 407± 25 | 28 ± 3 | 153± 13 | 69 ± 4 | 262 ± 15 |
|  | **Ideal** | 428 ± 29 | 29 ± 4 | 142 ± 16 | 70 ± 5 | 248 ± 18 |
|  | **P value** | 0.45 | **0.01** | 0.41 | 0.77 | 0.24 |
|  | | | | | | |
| **Cholesterol** | **Poor** | 430 ± 41 | 34 ± 5 | 152 ± 22 | 78 ± 7 | 279 ± 25 |
|  | **Intermediate** | 396 ± 21 | 33 ± 3 | 154 ± 11 | 68 ± 4 | 268 ± 13 |
|  | **Ideal** | 421 ± 20 | 30 ± 2 | 148 ± 11 | 70 ± 3 | 251 ± 12 |
|  | **P value** | 0.15 | 0.21 | 0.74 | **0.04** | **0.05** |
|  | | | | | | |
| **Glucose** | **Poor** | 365 ± 33 | 36 ± 4 | 168 ± 18 | 68 ± 6 | 300 ± 20 |
|  | **Intermediate** | 411 ± 32 | 35 ± 4 | 154 ± 17 | 73 ± 5 | 278 ± 19 |
|  | **Ideal** | 422 ± 17 | 29 ± 2 | 147 ± 9 | 70 ± 3 | 247 ± 10 |
|  | **P value** | **0.01** | **0.002** | 0.11 | 0.43 | **<0.001** |
|  | | | | | | |
| **Body Mass Index (BMI)** | **Poor** | 409 ± 18 | 33 ± 2 | 163 ± 9 | 75 ± 3 | 275 ± 11 |
|  | **Intermediate** | 414 ± 27 | 29 ± 3 | 137 ± 14 | 64 ± 4 | 249 ± 16 |
|  | **Ideal** | 416 ± 34 | 29 ± 4 | 131 ± 18 | 64 ± 6 | 231 ± 20 |
|  | **P value** | 0.91 | **0.02** | **<0.001** | **<0.001** | **<0.001** |
|  | | | | | | |
| **Exercise** | **Poor** | 392 ± 47 | 37 ± 6 | 165 ± 25 | 71 ± 8 | 267 ± 28 |
|  | **Intermediate** | 415 ± 24 | 34 ± 3 | 150 ± 13 | 72 ± 4 | 277 ± 14 |
|  | **Ideal** | 412 ± 18 | 29 ± 2 | 150 ± 9 | 69 ± 3 | 251 ± 11 |
|  | **P value** | 0.70 | **0.01** | 0.52 | 0.54 | **0.02** |
|  | | | | | | |
| **Diet** | **Poor** | 426 ± 24 | 29 ± 3 | 153 ± 13 | 72 ± 4 | 272 ± 14 |
|  | **Intermediate** | 405 ± 17 | 32 ± 2 | 149 ± 9 | 69 ± 3 | 256 ± 10 |
|  | **Ideal** | 399 ± 55 | 35 ± 7 | 160 ± 28 | 76 ± 9 | 260 ± 32 |
|  | **P value** | 0.33 | 0.20 | 0.73 | 0.20 | 0.19 |
|  | | | | | | |
| **Smoking** | **Poor** | 408.5 ± 128.8 | 33 ± 3 | 149 ± 15 | 74 ± 5 | 258 ± 17 |
|  | **Intermediate** | 340.5 ± 140.6 | 33 ± 8 | 155 ± 36 | 71 ± 11 | 279 ± 41 |
|  | **Ideal** | 416.1 ± 133.1 | 31 ± 2 | 152 ± 9 | 69 ± 3 | 261 ± 10 |
|  | **P value** | 0.11 | 0.57 | 0.93 | 0.16 | 0.64 |

**Table S5. Association of levels of five highlighted metabolites (glutamine, glutamate, alanine, tyrosine, urate) with LS7 score, adjusted for age, sex, and eGFR.**

| **Name** | **m/z_RT(sec)** | **Beta** | **Standard Error** | **p value** |
| --- | --- | --- | --- | --- |
| Alanine | mz90.055_t55.2 | -0.124 | 0.024 | 2.51E-07 |
| Glutamate | mz148.0605_t61.2 | -0.119 | 0.023 | 5.87E-07 |
| Urate | mz169.0359_t50.8 | -0.083 | 0.023 | 0.00032 |
| Tyrosine | mz182.0811_t49.6 | -0.076 | 0.024 | 0.0017 |
| Glutamine | mz147.0766_t70.5 | 0.03 | 0.024 | 0.0027 |

**Table S6. Pearson correlation analysis of levels of five highlighted metabolites.**

| **Metabolite 1** | **Metabolite 2** | **Correlation (CI), p value** |
| --- | --- | --- |
| mz147.0766_t70.5 | mz148.0605_t61.2 | 0.25 (0.15-0.34), 1.5e-06 |
| mz147.0766_t70.5 | mz169.0359_t50.8 | -0.26 (-0.35--0.16), 3.5e-07 |
| mz147.0766_t70.5 | mz182.0811_t49.6 | 0.35 (0.26-0.44), 1.6e-12 |
| mz147.0766_t70.5 | mz90.055_t55.2 | 0.39 (0.3-0.47), 3.6e-15 |
| mz148.0605_t61.2 | mz169.0359_t50.8 | 0.03 (-0.07-0.13), 5.4e-01 |
| mz148.0605_t61.2 | mz182.0811_t49.6 | 0.28 (0.18-0.37), 4.6e-08 |
| mz148.0605_t61.2 | mz90.055_t55.2 | 0.38 (0.29-0.47), 1.4e-1 |
| mz169.0359_t50.8 | mz182.0811_t49.6 | 0 (-0.1-0.1), 9.6e-01 |
| mz169.0359_t50.8 | mz90.055_t55.2 | -0.03 (-0.13-0.07), 5.3e-01 |
| mz182.0811_t49.6 | mz90.055_t55.2 | 0.44 (0.35-0.52), 5.9e-19 |

**Table S7: Multivariable linear regression demonstrated that in a model adjusting for age, sex, and eGFR, concentrations of all five of the metabolites were independently associated with LS7.**

| **Name** | **m/z_RT(sec)** | **Beta** | **Standard Error** | **p value** |
| --- | --- | --- | --- | --- |
| Glutamine | mz147.0766_t70.5 | 0.48 | 0.12 | <0.001 |
| Glutamate | mz148.0605_t61.2 | -0.33 | 0.11 | <0.001 |
| Urate | mz169.0359_t50.8 | -0.35 | 0.11 | <0.001 |
| Tyrosine | mz182.0811_t49.6 | -0.30 | 0.12 | 0.02 |
| Alanine | mz90.055_t55.2 | -0.53 | 0.12 | <0.001 |

**Figure S1: Analysis of metabolite activity network by *Mummichog* v2.0.6** (https://shuzhao-li.github.io/mummichog.org/)**.** The activity network combines analyses of metabolic pathways and metabolite modules (activity between metabolites).

***
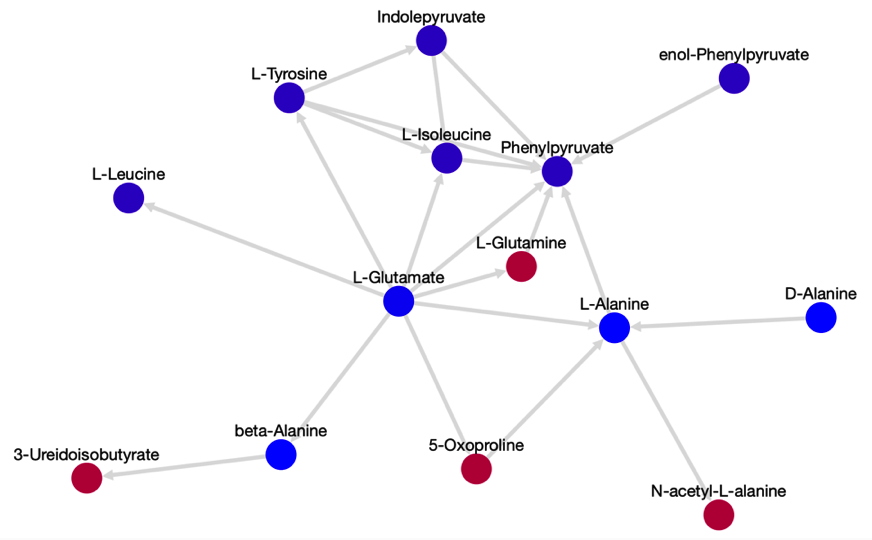
***
