# Supplementary material for: Metabolomic signatures of ideal cardiovascular health in black adults
Source: Sci Rep. 2024 Jan 20;14:1794. doi: 10.1038/s41598-024-51920-z (PMC10799852; doi:10.1038/s41598-024-51920-z)
Supplement: Supplementary file 2 — Supplementary Table S2. [file 41598_2024_51920_MOESM2_ESM.docx]

| Table S2: Metabolites from the metabolome wide association study (MWAS) that were significantly associated with ideal cardiovascular health as defined by AHA Life’s Simple 7 (LS7). | | | | | | | |
| --- | --- | --- | --- | --- | --- | --- | --- |
| m/z_RT(sec) | HMDB_Name | Beta | Standard Error | P-value | FDR (Q value) | HMDB Confidence | HMDB Chemical ID |
| mz293.5877_t57.7 | NA | -0.14 | 0.02 | 2.50E-09 | 0.00 | NA | NA |
| mz284.0804_t76.9 | NA | -0.14 | 0.02 | 7.05E-09 | 0.00 | NA | NA |
| mz293.0865_t64.4 | NA | -0.13 | 0.02 | 1.92E-08 | 0.00 | NA | NA |
| mz252.1078_t72.7 | Deoxyadenosine | -0.13 | 0.02 | 3.68E-08 | 0.00 | 2 | HMDB00101 |
| mz91.0583_t55.8 | 2-Methyl-1-propanethiol | -0.13 | 0.02 | 1.86E-07 | 0.00 | 2 | HMDB31245 |
| mz90.055_t55.2 | Alanine | -0.12 | 0.02 | 2.22E-07 | 0.00 | 2 | HMDB00056 |
| mz109.0285_t59.6 | Quinone | -0.12 | 0.02 | 7.82E-07 | 0.00 | 2 | HMDB03364 |
| mz149.064_t59.4 | L-Glutamic acid | -0.12 | 0.02 | 7.44E-07 | 0.00 | 3; 2 | HMDB00148 |
| mz148.0605_t61.2 | Glutamate | -0.12 | 0.02 | 8.51E-07 | 0.00 | 3 | HMDB00148 |
| mz204.0561_t57 | NA | -0.11 | 0.02 | 1.37E-06 | 0.00 | NA | NA |
| mz203.0529_t58.5 | NA | -0.11 | 0.02 | 1.96E-06 | 0.00 | NA | NA |
| mz813.617_t29.5 | NA | -0.11 | 0.02 | 9.56E-06 | 0.00 | NA | NA |
| mz182.0677_t30.4 | D-Glucose | -0.10 | 0.02 | 1.26E-05 | 0.01 | 3; 2 | HMDB00122 |
| mz468.3084_t31.3 | Buprenorphine | -0.10 | 0.02 | 2.15E-05 | 0.01 | 2 | HMDB10379 |
| mz330.9138_t49.9 | NA | -0.10 | 0.02 | 5.29E-06 | 0.00 | NA | NA |
| mz162.0219_t35.2 | NA | -0.10 | 0.02 | 2.43E-05 | 0.01 | NA | NA |
| mz250.9731_t51.3 | NA | -0.10 | 0.02 | 7.44E-06 | 0.00 | NA | NA |
| mz160.0254_t68.3 | (E)-4-Isothiocyanato-1-(methylthio)-1-butene | -0.10 | 0.02 | 2.19E-05 | 0.01 | 2 | HMDB31576 |
| mz128.0621_t23.2 | NA | -0.10 | 0.02 | 2.50E-05 | 0.01 | NA | NA |
| mz177.0684_t36.9 | NA | -0.10 | 0.02 | 2.97E-05 | 0.01 | NA | NA |
| mz331.1317_t57.1 | NA | -0.10 | 0.02 | 3.90E-05 | 0.01 | NA | NA |
| mz350.895_t42.2 | NA | -0.10 | 0.02 | 1.49E-05 | 0.01 | NA | NA |
| mz338.9455_t52.2 | NA | -0.10 | 0.02 | 1.79E-05 | 0.01 | NA | NA |
| mz469.3118_t31.6 | NA | -0.10 | 0.02 | 4.58E-05 | 0.01 | NA | NA |
| mz524.8476_t52 | NA | -0.10 | 0.02 | 5.61E-05 | 0.01 | NA | NA |
| mz294.1546_t43.1 | N-(1-Deoxy-1-fructosyl)leucine | -0.10 | 0.02 | 5.42E-05 | 0.01 | 2 | HMDB37840 |
| mz325.1114_t71.6 | Melibiose | -0.10 | 0.02 | 6.06E-05 | 0.01 | 3 | HMDB00048 |
| mz212.9995_t52.2 | NA | -0.10 | 0.02 | 1.77E-05 | 0.01 | NA | NA |
| mz328.9165_t49.5 | NA | -0.10 | 0.02 | 2.04E-05 | 0.01 | NA | NA |
| mz270.958_t51.5 | NA | -0.10 | 0.02 | 1.89E-05 | 0.01 | NA | NA |
| mz269.2262_t23.4 | NA | -0.10 | 0.02 | 5.73E-05 | 0.01 | NA | NA |
| mz201.1637_t22.9 | (S)-gamma-Calacorene | -0.10 | 0.02 | 6.24E-05 | 0.01 | 2 | HMDB36451 |
| mz270.2295_t23.2 | NA | -0.10 | 0.02 | 6.24E-05 | 0.01 | NA | NA |
| mz139.5122_t58.7 | NA | -0.09 | 0.02 | 9.43E-05 | 0.02 | NA | NA |
| mz183.5391_t60.7 | NA | -0.09 | 0.02 | 9.34E-05 | 0.02 | NA | NA |
| mz812.6129_t27.3 | PC(16:1(9Z)22:2(13Z16Z)) | -0.09 | 0.02 | 8.65E-05 | 0.02 | 2 | HMDB08020 |
| mz246.1697_t28.3 | 2-Methylbutyroylcarnitine | -0.09 | 0.02 | 3.23E-05 | 0.01 | 3 | HMDB00378 |
| mz157.1012_t23.1 | 26-Dimethyl-naphtalene | -0.09 | 0.02 | 9.49E-05 | 0.02 | 2 | HMDB59764 |
| mz369.6788_t31.4 | NA | -0.09 | 0.02 | 0.00011799 | 0.02 | NA | NA |
| mz304.1013_t47.7 | Gynocardin | -0.09 | 0.02 | 0.0001115 | 0.02 | 2 | HMDB29913 |
| mz202.5526_t58.4 | NA | -0.09 | 0.02 | 0.00010237 | 0.02 | NA | NA |
| mz388.8731_t50.2 | NA | -0.09 | 0.02 | 4.13E-05 | 0.01 | NA | NA |
| mz222.0057_t48.1 | NA | -0.09 | 0.02 | 9.88E-05 | 0.02 | NA | NA |
| mz444.8341_t51.2 | NA | -0.09 | 0.02 | 4.74E-05 | 0.01 | NA | NA |
| mz248.9761_t51.3 | NA | -0.09 | 0.02 | 5.02E-05 | 0.01 | NA | NA |
| mz814.6267_t34.5 | PC(14:1(9Z)24:1(15Z)) | -0.09 | 0.02 | 0.00013856 | 0.02 | 3 | HMDB07927 |
| mz337.923_t49.3 | NA | -0.09 | 0.02 | 0.00010744 | 0.02 | NA | NA |
| mz125.9642_t190.4 | NA | -0.09 | 0.02 | 0.00017538 | 0.02 | NA | NA |
| mz203.5558_t62.6 | NA | -0.09 | 0.02 | 0.00017865 | 0.02 | NA | NA |
| mz213.1637_t22.8 | 26-Diisopropylnaphthalene | -0.09 | 0.02 | 0.00016918 | 0.02 | 2 | HMDB59902 |
| mz189.1637_t23.2 | 1-Methyl-13-cyclohexadiene | -0.09 | 0.02 | 0.00016501 | 0.02 | 2 | HMDB31532 |
| mz446.8308_t50.7 | NA | -0.09 | 0.02 | 7.16E-05 | 0.01 | NA | NA |
| mz547.3556_t29.4 | LysoPC(20:3(5Z8Z11Z)); LysoPC(20:3(8Z11Z14Z)) | -0.09 | 0.02 | 0.00025249 | 0.03 | 3 | HMDB10393 |
| mz343.9586_t47.9 | NA | -0.09 | 0.02 | 0.00026772 | 0.03 | NA | NA |
| mz173.1323_t23.5 | 12-Dihydro-116-trimethylnaphthalene | -0.09 | 0.02 | 0.00025116 | 0.03 | 2 | HMDB40284 |
| mz169.0359_t50.8 | Urate | -0.09 | 0.02 | 0.00018068 | 0.02 | 2 | HMDB00289 |
| mz852.5416_t48.6 | NA | -0.09 | 0.02 | 0.0002875 | 0.03 | NA | NA |
| mz335.9258_t49.3 | NA | -0.09 | 0.02 | 0.00022436 | 0.03 | NA | NA |
| mz755.5451_t26.4 | NA | -0.09 | 0.02 | 0.00029057 | 0.03 | NA | NA |
| mz176.0659_t38.7 | N-amidino-L-aspartate | -0.09 | 0.02 | 0.00036943 | 0.04 | 2 | HMDB03157 |
| mz234.9813_t47.2 | NA | -0.09 | 0.02 | 0.00013525 | 0.02 | NA | NA |
| mz152.002_t73.2 | NA | -0.09 | 0.02 | 0.00031718 | 0.03 | NA | NA |
| mz402.3452_t23 | 7-Ketocholesterol | -0.09 | 0.02 | 0.00037083 | 0.04 | 3 | HMDB00501 |
| mz280.9863_t53.8 | NA | -0.09 | 0.02 | 0.0001428 | 0.02 | NA | NA |
| mz87.0998_t38.8 | Piperidine | -0.09 | 0.02 | 0.00021225 | 0.03 | 3 | HMDB34301 |
| mz86.0965_t38.9 | Neurine; Piperidine | -0.08 | 0.02 | 0.0002504 | 0.03 | 3 | HMDB31259 |
| mz362.0201_t51.6 | NA | -0.08 | 0.02 | 0.00052103 | 0.05 | NA | NA |
| mz305.2472_t23.2 | Arachidonic acid | -0.08 | 0.02 | 0.00053472 | 0.05 | 3 | HMDB01043 |
| mz400.342_t22.4 | L-Palmitoylcarnitine | -0.08 | 0.02 | 0.0004918 | 0.05 | 2 | HMDB00222 |
| mz221.0364_t69.8 | NA | -0.08 | 0.02 | 0.00047626 | 0.05 | NA | NA |
| mz459.8771_t48.9 | NA | -0.08 | 0.02 | 0.00060777 | 0.05 | NA | NA |
| mz292.9399_t52.2 | NA | -0.08 | 0.02 | 0.00023006 | 0.03 | NA | NA |
| mz224.1128_t56.5 | NA | -0.08 | 0.02 | 0.00063227 | 0.06 | NA | NA |
| mz466.8871_t43.4 | NA | -0.08 | 0.02 | 0.00059423 | 0.05 | NA | NA |
| mz115.0155_t263 | NA | -0.08 | 0.02 | 0.00076846 | 0.06 | NA | NA |
| mz190.0035_t289.7 | NA | -0.08 | 0.02 | 0.00074622 | 0.06 | NA | NA |
| mz113.9637_t285.6 | NA | -0.08 | 0.02 | 0.00077061 | 0.06 | NA | NA |
| mz272.9576_t54.5 | NA | -0.08 | 0.02 | 0.00037183 | 0.04 | NA | NA |
| mz546.3546_t28 | LysoPC(20:3(5Z8Z11Z)) | -0.08 | 0.02 | 0.00080223 | 0.06 | 3 | HMDB10393 |
| mz195.0655_t59.4 | Ferulate | -0.08 | 0.02 | 0.00072381 | 0.06 | 2 | HMDB00954 |
| mz163.0603_t61 | 2-Hydroxyadipic acid | -0.08 | 0.02 | 0.00074357 | 0.06 | 2 | HMDB00321 |
| mz187.1481_t23.1 | 7-Ethyl-56-dihydro-14-dimethylazulene | -0.08 | 0.02 | 0.00066472 | 0.06 | 2 | HMDB35759 |
| mz302.9689_t51.4 | NA | -0.08 | 0.02 | 0.00059387 | 0.05 | NA | NA |
| mz171.1168_t22.7 | 167-Trimethylnaphthalene; 145-Trimethyl-naphtalene | -0.08 | 0.02 | 0.0008043 | 0.06 | 2 | HMDB59701 |
| mz464.1907_t33.5 | Dihydroisomorphine-3-glucuronide | -0.08 | 0.02 | 0.00088155 | 0.07 | 3 | HMDB60820 |
| mz338.0519_t90.4 | Famotidine | -0.08 | 0.02 | 0.00084235 | 0.06 | 2 | HMDB01919 |
| mz534.8042_t50.4 | NA | -0.08 | 0.02 | 0.00067962 | 0.06 | NA | NA |
| mz275.1345_t53.1 | Glutaminyl-Glutamine | -0.08 | 0.02 | 0.00095013 | 0.07 | 2 | HMDB28795 |
| mz320.0043_t173.7 | NA | -0.08 | 0.02 | 0.00060863 | 0.05 | NA | NA |
| mz432.9186_t48.3 | NA | -0.08 | 0.02 | 0.00098031 | 0.07 | NA | NA |
| mz386.8744_t45.3 | NA | -0.08 | 0.02 | 0.00082921 | 0.06 | NA | NA |
| mz490.2892_t32.1 | NA | -0.08 | 0.02 | 0.000991 | 0.07 | NA | NA |
| mz134.1085_t38.9 | L-Isoleucine | -0.08 | 0.02 | 0.00058034 | 0.05 | 3 | HMDB00172 |
| mz471.9011_t48.4 | NA | -0.08 | 0.02 | 0.00085407 | 0.06 | NA | NA |
| mz134.1061_t38.7 | L-Isoleucine | -0.08 | 0.02 | 0.00065492 | 0.06 | 3 | HMDB00172 |
| mz309.8917_t55 | NA | -0.08 | 0.02 | 0.00108734 | 0.08 | NA | NA |
| mz837.6137_t31.2 | NA | -0.08 | 0.02 | 0.00105038 | 0.07 | NA | NA |
| mz133.1052_t38.8 | L-Isoleucine | -0.08 | 0.02 | 0.00074131 | 0.06 | 3 | HMDB00172 |
| mz732.5531_t25.2 | PC(14:018:1(11Z)) | -0.08 | 0.02 | 0.0012685 | 0.08 | 2 | HMDB07872 |
| mz175.1481_t23.2 | 57alpha-Dihydro-1447a-tetramethyl-4H-indene | -0.08 | 0.02 | 0.00105305 | 0.07 | 2 | HMDB36683 |
| mz136.0757_t48.6 | N-Acetylarylamine | -0.08 | 0.02 | 0.00136851 | 0.09 | 3 | HMDB01250 |
| mz360.9273_t50.4 | NA | -0.08 | 0.02 | 0.00063671 | 0.06 | NA | NA |
| mz220.0087_t49 | NA | -0.08 | 0.02 | 0.00099993 | 0.07 | NA | NA |
| mz705.7332_t48.7 | NA | -0.08 | 0.02 | 0.00129969 | 0.08 | NA | NA |
| mz501.0302_t52.7 | NA | -0.08 | 0.02 | 0.0013907 | 0.09 | NA | NA |
| mz132.1019_t39.1 | Leucine/Isoleucine | -0.08 | 0.02 | 0.00085758 | 0.06 | 3 | HMDB00172 |
| mz117.0676_t62.5 | L-Proline | -0.08 | 0.02 | 0.00143287 | 0.09 | 3 | HMDB00162 |
| mz450.013_t46.1 | Adenosine phosphosulfate | -0.08 | 0.02 | 0.0015235 | 0.09 | 3 | HMDB01003 |
| mz438.943_t53 | NA | -0.08 | 0.02 | 0.00148837 | 0.09 | NA | NA |
| mz294.5892_t58.2 | NA | -0.08 | 0.02 | 0.00146796 | 0.09 | NA | NA |
| mz593.8555_t47.9 | NA | -0.08 | 0.02 | 0.00160562 | 0.09 | NA | NA |
| mz743.8552_t52.6 | NA | -0.08 | 0.02 | 0.00165007 | 0.09 | NA | NA |
| mz287.2366_t23.3 | Vitamin A | -0.08 | 0.02 | 0.00180986 | 0.10 | 2 | HMDB00305 |
| mz117.074_t58.8 | L-Proline | -0.08 | 0.02 | 0.00175454 | 0.10 | 3 | HMDB00162 |
| mz336.5864_t267.2 | NA | -0.08 | 0.02 | 0.00162745 | 0.09 | NA | NA |
| mz336.8516_t210.8 | NA | -0.08 | 0.02 | 0.00188695 | 0.10 | NA | NA |
| mz176.0705_t24.9 | Indole-3-acetate | -0.08 | 0.02 | 0.00172752 | 0.10 | 2 | HMDB00197 |
| mz824.7499_t51.6 | NA | -0.08 | 0.02 | 0.00194091 | 0.10 | NA | NA |
| mz199.148_t23.1 | 7-Isopropyl-14-dimethylazulene | -0.08 | 0.02 | 0.00187326 | 0.10 | 2 | HMDB36648 |
| mz642.8441_t53.9 | NA | -0.08 | 0.02 | 0.00189271 | 0.10 | NA | NA |
| mz134.5464_t85.7 | NA | -0.08 | 0.02 | 0.00198594 | 0.10 | NA | NA |
| mz116.0707_t60.4 | Proline | -0.08 | 0.02 | 0.00199084 | 0.10 | 3 | HMDB00162 |
| mz491.293_t32.2 | NA | -0.08 | 0.02 | 0.00205631 | 0.10 | NA | NA |
| mz347.9519_t49.2 | NA | -0.07 | 0.02 | 0.00187536 | 0.10 | NA | NA |
| mz587.9695_t46.1 | Adenosine tetraphosphate | -0.07 | 0.02 | 0.00208738 | 0.11 | 2 | HMDB01364 |
| mz401.9165_t45.1 | NA | -0.07 | 0.02 | 0.00222014 | 0.11 | NA | NA |
| mz150.9593_t39.6 | NA | -0.07 | 0.02 | 0.0022277 | 0.11 | NA | NA |
| mz95.0855_t23.6 | 1-Methyl-13-cyclohexadiene | -0.07 | 0.02 | 0.00199467 | 0.10 | 2 | HMDB31532 |
| mz341.9621_t48.6 | NA | -0.07 | 0.02 | 0.00229224 | 0.11 | NA | NA |
| mz337.949_t33.1 | NA | -0.07 | 0.02 | 0.0022657 | 0.11 | NA | NA |
| mz262.8471_t252.2 | NA | -0.07 | 0.02 | 0.00216091 | 0.11 | NA | NA |
| mz359.0594_t39.1 | Etoricoxib | -0.07 | 0.02 | 0.0023572 | 0.11 | 2 | HMDB15565 |
| mz517.3094_t31.4 | LysoPC(18:4(6Z9Z12Z15Z)) | -0.07 | 0.02 | 0.00243373 | 0.12 | 3 | HMDB10389 |
| mz610.184_t22.8 | Peonidin 3-rhamnoside 5-glucoside | -0.07 | 0.02 | 0.00253422 | 0.12 | 3 | HMDB38090 |
| mz370.9541_t172.7 | NA | -0.07 | 0.02 | 0.00254979 | 0.12 | NA | NA |
| mz403.9132_t43.9 | NA | -0.07 | 0.02 | 0.00235022 | 0.11 | NA | NA |
| mz346.9986_t57.8 | NA | -0.07 | 0.02 | 0.00261119 | 0.12 | NA | NA |
| mz270.9776_t39.8 | NA | -0.07 | 0.02 | 0.00257066 | 0.12 | NA | NA |
| mz218.1385_t31.2 | Propionylcarnitine | -0.07 | 0.02 | 0.00159082 | 0.09 | 2 | HMDB00824 |
| mz148.9612_t39.5 | NA | -0.07 | 0.02 | 0.00250718 | 0.12 | NA | NA |
| mz182.0811_t49.6 | Tyrosine | -0.07 | 0.02 | 0.00266078 | 0.12 | 3 | HMDB00158 |
| mz123.0441_t49.6 | 3-Hydroxybenzaldehyde | -0.07 | 0.02 | 0.00263031 | 0.12 | 2 | HMDB01870 |
| mz258.128_t72.2 | NA | -0.07 | 0.02 | 0.00259475 | 0.12 | NA | NA |
| mz290.0759_t58.1 | NA | -0.07 | 0.02 | 0.00242814 | 0.12 | NA | NA |
| mz836.6129_t33.5 | PC(18:022:5(4Z7Z10Z13Z16Z)) | -0.07 | 0.02 | 0.00244772 | 0.12 | 2 | HMDB08055 |
| mz643.2882_t33.5 | NA | -0.07 | 0.02 | 0.00279252 | 0.12 | NA | NA |
| mz319.9736_t175.1 | NA | -0.07 | 0.02 | 0.00275229 | 0.12 | NA | NA |
| mz216.0633_t88.4 | Glycerylphosphorylethanolamine | -0.07 | 0.02 | 0.00279774 | 0.12 | 2 | HMDB00114 |
| mz147.1168_t23.7 | NA | -0.07 | 0.02 | 0.00264431 | 0.12 | NA | NA |
| mz226.0475_t57.6 | Furazolidone | -0.07 | 0.02 | 0.00289286 | 0.12 | 3 | HMDB14752 |
| mz270.0639_t79.6 | NA | -0.07 | 0.02 | 0.00270468 | 0.12 | NA | NA |
| mz185.0441_t61.2 | 34-Dihydroxymandelic acid | -0.07 | 0.02 | 0.00282764 | 0.12 | 2 | HMDB01866 |
| mz446.9122_t40.4 | NA | -0.07 | 0.02 | 0.00290788 | 0.12 | NA | NA |
| mz285.8133_t90 | NA | -0.07 | 0.02 | 0.00294361 | 0.12 | NA | NA |
| mz838.6212_t43.6 | NA | -0.07 | 0.02 | 0.00288242 | 0.12 | NA | NA |
| mz284.0036_t48.7 | NA | -0.07 | 0.02 | 0.00311151 | 0.13 | NA | NA |
| mz389.9033_t245 | NA | -0.07 | 0.02 | 0.00310177 | 0.13 | NA | NA |
| mz232.1541_t30.8 | Isobutyryl-L-carnitine | -0.07 | 0.02 | 0.00273318 | 0.12 | 3 | HMDB00736 |
| mz312.0423_t48.9 | NA | -0.07 | 0.02 | 0.00317072 | 0.13 | NA | NA |
| mz269.1381_t24.5 | Kamahine C | -0.07 | 0.02 | 0.00332363 | 0.13 | 2 | HMDB38935 |
| mz132.9871_t42.1 | NA | -0.07 | 0.02 | 0.00329082 | 0.13 | NA | NA |
| mz344.9116_t49.1 | NA | -0.07 | 0.02 | 0.00194903 | 0.10 | NA | NA |
| mz457.8828_t36.6 | NA | -0.07 | 0.02 | 0.00348762 | 0.13 | NA | NA |
| mz594.1581_t280.4 | NA | -0.07 | 0.02 | 0.00349496 | 0.13 | NA | NA |
| mz329.1423_t37.8 | 7-hydroxyolanzapine | -0.07 | 0.02 | 0.00312152 | 0.13 | 2 | HMDB60958 |
| mz522.9325_t53.6 | NA | -0.07 | 0.02 | 0.00333347 | 0.13 | NA | NA |
| mz161.0185_t35.2 | NA | -0.07 | 0.02 | 0.00344243 | 0.13 | NA | NA |
| mz91.0543_t37 | NA | -0.07 | 0.02 | 0.00315833 | 0.13 | NA | NA |
| mz444.9154_t53.8 | NA | -0.07 | 0.02 | 0.00332209 | 0.13 | NA | NA |
| mz298.0249_t48.6 | NA | -0.07 | 0.02 | 0.0033797 | 0.13 | NA | NA |
| mz165.0546_t49.3 | Phenylpuruvate | -0.07 | 0.02 | 0.00362507 | 0.14 | 3 | HMDB00205 |
| mz280.139_t48 | N-(1-Deoxy-1-fructosyl)valine | -0.07 | 0.02 | 0.00340234 | 0.13 | 2 | HMDB37844 |
| mz260.9487_t195.8 | NA | -0.07 | 0.02 | 0.00385908 | 0.14 | NA | NA |
| mz763.9134_t43.6 | NA | -0.07 | 0.02 | 0.00393755 | 0.14 | NA | NA |
| mz167.0557_t44.5 | Arabinonic acid | -0.07 | 0.02 | 0.00394382 | 0.14 | 2 | HMDB00539 |
| mz417.3723_t22.7 | Gamma-Tocopherol | -0.07 | 0.02 | 0.00388849 | 0.14 | 2 | HMDB01492 |
| mz294.0324_t48.1 | NA | -0.07 | 0.02 | 0.00411414 | 0.15 | NA | NA |
| mz473.8974_t50.7 | NA | -0.07 | 0.02 | 0.00367638 | 0.14 | NA | NA |
| mz166.058_t48.3 | Phenylpyruvic acid | -0.07 | 0.02 | 0.00390053 | 0.14 | 3 | HMDB00205 |
| mz989.6549_t31.3 | NA | -0.07 | 0.02 | 0.00430485 | 0.15 | NA | NA |
| mz603.0207_t46.6 | NA | -0.07 | 0.02 | 0.00433827 | 0.15 | NA | NA |
| mz495.3277_t30.9 | LysoPC(16:1(9Z)) | -0.07 | 0.02 | 0.00442412 | 0.16 | 3 | HMDB10383 |
| mz117.9699_t74.8 | NA | -0.07 | 0.02 | 0.00448381 | 0.16 | NA | NA |
| mz124.9911_t83.8 | NA | -0.07 | 0.02 | 0.00452331 | 0.16 | NA | NA |
| mz262.1649_t35.3 | NA | -0.07 | 0.02 | 0.00371521 | 0.14 | NA | NA |
| mz321.9696_t48 | NA | -0.07 | 0.02 | 0.00493989 | 0.16 | NA | NA |
| mz494.3243_t29.5 | LysoPC(16:1(9Z)) | -0.07 | 0.02 | 0.00494316 | 0.16 | 3 | HMDB10383 |
| mz287.9956_t48.3 | NA | -0.07 | 0.02 | 0.0046657 | 0.16 | NA | NA |
| mz591.1072_t61.4 | NA | -0.07 | 0.02 | 0.00471528 | 0.16 | NA | NA |
| mz592.7625_t51.6 | NA | -0.07 | 0.02 | 0.00422003 | 0.15 | NA | NA |
| mz118.0751_t55.8 | L-Proline | -0.07 | 0.02 | 0.00508874 | 0.17 | 3 | HMDB00162 |
| mz139.5311_t240.5 | NA | -0.07 | 0.02 | 0.00507436 | 0.17 | NA | NA |
| mz582.806_t52.3 | NA | -0.07 | 0.02 | 0.00365965 | 0.14 | NA | NA |
| mz287.2028_t31.6 | 2-Octenoylcarnitine | -0.07 | 0.02 | 0.00342237 | 0.13 | 3; 2 | HMDB13324 |
| mz172.0671_t72.9 | NA | -0.07 | 0.02 | 0.00544954 | 0.18 | NA | NA |
| mz733.5562_t26.1 | NA | -0.07 | 0.02 | 0.00550459 | 0.18 | NA | NA |
| mz658.6818_t51.3 | NA | -0.07 | 0.02 | 0.00516965 | 0.17 | NA | NA |
| mz271.2417_t24.8 | ar-Artemisene | -0.07 | 0.02 | 0.005332 | 0.17 | 2 | HMDB39155 |
| mz128.9711_t285.5 | NA | -0.07 | 0.02 | 0.0056166 | 0.18 | NA | NA |
| mz213.1596_t25.3 | 14-Bipiperidine-1-carboxylic acid | -0.07 | 0.02 | 0.00594149 | 0.18 | 2 | HMDB60336 |
| mz492.812_t57.6 | NA | -0.07 | 0.02 | 0.00600344 | 0.18 | NA | NA |
| mz554.7995_t51.6 | NA | -0.07 | 0.02 | 0.0059508 | 0.18 | NA | NA |
| mz119.0492_t46.3 | 246-Octatriyn-1-ol | -0.07 | 0.02 | 0.00604239 | 0.18 | 2 | HMDB30968 |
| mz399.3466_t207.6 | NA | -0.07 | 0.02 | 0.00610457 | 0.18 | NA | NA |
| mz211.1439_t27.5 | LL-Cyclo(leucylprolyl) | -0.07 | 0.02 | 0.00570534 | 0.18 | 3 | HMDB34276 |
| mz409.9489_t50.1 | NA | -0.07 | 0.02 | 0.0062303 | 0.19 | NA | NA |
| mz140.9911_t50.7 | NA | -0.07 | 0.02 | 0.00585643 | 0.18 | NA | NA |
| mz178.024_t48.8 | NA | -0.07 | 0.02 | 0.00635248 | 0.19 | NA | NA |
| mz191.9775_t55 | NA | -0.07 | 0.02 | 0.00648275 | 0.19 | NA | NA |
| mz282.1189_t54.1 | 1-Methyladenosine | -0.07 | 0.02 | 0.00602235 | 0.18 | 2 | HMDB03331 |
| mz612.9192_t54.9 | NA | -0.07 | 0.02 | 0.0064478 | 0.19 | NA | NA |
| mz296.5591_t60.4 | NA | -0.07 | 0.02 | 0.00654706 | 0.19 | NA | NA |
| mz342.1394_t56.8 | Lactosamine | -0.07 | 0.02 | 0.00646758 | 0.19 | 2 | HMDB06591 |
| mz145.9801_t167.9 | NA | -0.07 | 0.02 | 0.00671481 | 0.19 | NA | NA |
| mz125.9536_t271.2 | NA | -0.07 | 0.02 | 0.00669419 | 0.19 | NA | NA |
| mz402.8702_t48.9 | NA | -0.07 | 0.02 | 0.00607328 | 0.18 | NA | NA |
| mz201.045_t59.4 | NA | -0.07 | 0.02 | 0.00665441 | 0.19 | NA | NA |
| mz566.7422_t51.1 | NA | -0.07 | 0.02 | 0.00583461 | 0.18 | NA | NA |
| mz375.9675_t174.8 | NA | -0.07 | 0.02 | 0.00549216 | 0.18 | NA | NA |
| mz701.9573_t47.4 | NA | -0.07 | 0.02 | 0.00715113 | 0.20 | NA | NA |
| mz159.1168_t23.5 | Butadiene-styrene rubber | -0.07 | 0.02 | 0.00619189 | 0.19 | 2 | HMDB32178 |
| mz332.9142_t41.1 | NA | -0.07 | 0.02 | 0.0066553 | 0.19 | NA | NA |
| mz335.0915_t62.4 | NA | -0.07 | 0.02 | 0.00703012 | 0.20 | NA | NA |
| mz428.9145_t52.4 | NA | -0.07 | 0.02 | 0.00605592 | 0.18 | NA | NA |
| mz370.9561_t52.2 | NA | -0.07 | 0.02 | 0.00669777 | 0.19 | NA | NA |
| mz425.0998_t54.3 | NA | -0.07 | 0.02 | 0.00719633 | 0.20 | NA | NA |
| mz589.8158_t49 | NA | -0.07 | 0.02 | 0.00694127 | 0.19 | NA | NA |
| mz126.5272_t101.2 | NA | -0.06 | 0.02 | 0.0062336 | 0.19 | NA | NA |
| mz102.0914_t44.4 | 5-Aminopentanal | -0.06 | 0.02 | 0.00650911 | 0.19 | 2 | HMDB12815 |
| mz454.8606_t46.3 | NA | -0.06 | 0.02 | 0.00716175 | 0.20 | NA | NA |
| mz834.5932_t31.8 | PC(18:022:6(4Z7Z10Z13Z16Z19Z)) | -0.06 | 0.02 | 0.00625779 | 0.19 | 2 | HMDB08057 |
| mz309.1291_t86.4 | NA | -0.06 | 0.02 | 0.00728377 | 0.20 | NA | NA |
| mz152.0221_t35.9 | NA | 0.06 | 0.02 | 0.00676571 | 0.19 | NA | NA |
| mz771.6081_t28.5 | NA | 0.07 | 0.02 | 0.00715296 | 0.20 | NA | NA |
| mz135.9262_t63.6 | NA | 0.07 | 0.02 | 0.0071847 | 0.20 | NA | NA |
| mz770.6019_t27.9 | PC(14:022:1(13Z)) | 0.07 | 0.02 | 0.00730789 | 0.20 | 3; 2 | HMDB07887 |
| mz449.0952_t58.5 | NA | 0.07 | 0.02 | 0.00670842 | 0.19 | NA | NA |
| mz447.0985_t56.4 | NA | 0.07 | 0.02 | 0.00659583 | 0.19 | NA | NA |
| mz175.1117_t279.7 | 6-Phenyl-3-hexen-2-one | 0.07 | 0.02 | 0.00643432 | 0.19 | 2 | HMDB31622 |
| mz149.0811_t69.7 | L-Glutamine | 0.07 | 0.02 | 0.00588372 | 0.18 | 3; 2 | HMDB00641 |
| mz131.1068_t278.3 | Heptanoic acid | 0.07 | 0.02 | 0.0054923 | 0.18 | 2 | HMDB00666 |
| mz116.0343_t74.3 | Maleamate | 0.07 | 0.02 | 0.00568677 | 0.18 | NA | NA |
| mz821.6241_t27.7 | NA | 0.07 | 0.02 | 0.00515147 | 0.17 | NA | NA |
| mz549.828_t49.7 | NA | 0.07 | 0.02 | 0.00573059 | 0.18 | NA | NA |
| mz413.7656_t27.8 | NA | 0.07 | 0.02 | 0.00550146 | 0.18 | NA | NA |
| mz242.175_t201.2 | Dioscoretine | 0.07 | 0.02 | 0.00542038 | 0.18 | 2 | HMDB38588 |
| mz130.05_t65.3 | Oxoproline | 0.07 | 0.02 | 0.0049599 | 0.16 | 3 | HMDB00267 |
| mz265.5935_t60 | NA | 0.07 | 0.02 | 0.00486459 | 0.16 | NA | NA |
| mz502.3269_t29.8 | NA | 0.07 | 0.02 | 0.00487102 | 0.16 | NA | NA |
| mz199.119_t57.7 | NA | 0.07 | 0.02 | 0.00473435 | 0.16 | NA | NA |
| mz133.9269_t69.7 | NA | 0.07 | 0.02 | 0.00456878 | 0.16 | NA | NA |
| mz299.6085_t58.1 | NA | 0.07 | 0.02 | 0.00457748 | 0.16 | NA | NA |
| mz289.0801_t78.6 | NA | 0.07 | 0.02 | 0.00446356 | 0.16 | NA | NA |
| mz175.1078_t58.9 | N-Acetylornithine | 0.07 | 0.02 | 0.00371855 | 0.14 | 2 | HMDB03357 |
| mz812.6715_t31.5 | NA | 0.07 | 0.02 | 0.0040022 | 0.14 | NA | NA |
| mz159.0841_t69.5 | S-Isopropyl 3-methylbut-2-enethioate | 0.07 | 0.02 | 0.00410103 | 0.15 | 2 | HMDB32354 |
| mz615.819_t52.6 | NA | 0.07 | 0.02 | 0.00397622 | 0.14 | NA | NA |
| mz391.2704_t34.9 | NA | 0.07 | 0.02 | 0.0037961 | 0.14 | NA | NA |
| mz131.0535_t68.5 | Pyroglutamic acid | 0.07 | 0.02 | 0.00346202 | 0.13 | 3; 2 | HMDB00267 |
| mz813.6836_t34.1 | SM(d18:124:1(15Z)) | 0.07 | 0.02 | 0.00360202 | 0.14 | 2 | HMDB12107 |
| mz147.0766_t70.5 | Glutamine | 0.07 | 0.02 | 0.00334192 | 0.13 | 3 | HMDB00267 |
| mz428.8265_t50.8 | NA | 0.07 | 0.02 | 0.00332611 | 0.13 | NA | NA |
| mz587.2772_t23.1 | NA | 0.07 | 0.02 | 0.00288577 | 0.12 | NA | NA |
| mz351.0993_t43.2 | Penicillin V | 0.07 | 0.02 | 0.0028727 | 0.12 | 2 | HMDB14561 |
| mz152.0321_t59.9 | NA | 0.07 | 0.02 | 0.00246937 | 0.12 | NA | NA |
| mz390.7706_t34.5 | NA | 0.07 | 0.02 | 0.00251601 | 0.12 | NA | NA |
| mz386.9429_t57.2 | NA | 0.07 | 0.02 | 0.00238057 | 0.11 | NA | NA |
| mz597.9094_t35.8 | NA | 0.08 | 0.02 | 0.00197581 | 0.10 | NA | NA |
| mz814.6872_t35.2 | NA | 0.08 | 0.02 | 0.00197885 | 0.10 | NA | NA |
| mz564.7975_t44.2 | NA | 0.08 | 0.02 | 0.00183189 | 0.10 | NA | NA |
| mz298.2457_t21.9 | NA | 0.08 | 0.02 | 0.00171157 | 0.10 | NA | NA |
| mz549.4091_t22.8 | 3-Hydroxy-ee-caroten-3-one | 0.08 | 0.02 | 0.00136936 | 0.09 | 3 | HMDB02020 |
| mz135.5322_t56.4 | NA | 0.08 | 0.02 | 0.00159085 | 0.09 | NA | NA |
| mz260.8974_t43.6 | NA | 0.08 | 0.02 | 0.00145003 | 0.09 | NA | NA |
| mz142.5225_t60.9 | NA | 0.08 | 0.02 | 0.00147404 | 0.09 | NA | NA |
| mz101.071_t69.7 | N-Nitroso-pyrrolidine | 0.08 | 0.02 | 0.00133981 | 0.09 | 2 | HMDB31642 |
| mz457.232_t33.8 | 1-Lyso-2-arachidonoyl-phosphatidate | 0.08 | 0.02 | 0.00127446 | 0.08 | 2 | HMDB12496 |
| mz208.9819_t35.6 | NA | 0.08 | 0.02 | 0.00143316 | 0.09 | NA | NA |
| mz586.3938_t24 | NA | 0.08 | 0.02 | 0.00137844 | 0.09 | NA | NA |
| mz130.1056_t69.8 | NA | 0.08 | 0.02 | 0.00130049 | 0.08 | NA | NA |
| mz440.8702_t26.6 | NA | 0.08 | 0.02 | 0.00116269 | 0.08 | NA | NA |
| mz98.9985_t72.2 | NA | 0.08 | 0.02 | 0.00122539 | 0.08 | NA | NA |
| mz742.574_t27.4 | PC(16:1(9Z)P-18:1(11Z)) | 0.08 | 0.02 | 0.00115009 | 0.08 | 2 | HMDB08029 |
| mz445.2741_t23.6 | NA | 0.08 | 0.02 | 0.00119258 | 0.08 | NA | NA |
| mz689.5589_t28.5 | NA | 0.08 | 0.02 | 0.00083502 | 0.06 | NA | NA |
| mz727.7183_t52.6 | NA | 0.08 | 0.02 | 0.00093187 | 0.07 | NA | NA |
| mz744.5877_t29.8 | PC(16:0P-18:1(11Z)) | 0.08 | 0.02 | 0.00062506 | 0.06 | 2 | HMDB07996 |
| mz445.9597_t39.1 | NA | 0.08 | 0.02 | 0.00054425 | 0.05 | NA | NA |
| mz384.3222_t29 | NA | 0.09 | 0.02 | 0.00047141 | 0.05 | NA | NA |
| mz747.6121_t34.3 | NA | 0.09 | 0.02 | 0.00038062 | 0.04 | NA | NA |
| mz745.5914_t27.3 | NA | 0.09 | 0.02 | 0.00030054 | 0.03 | NA | NA |
| mz552.4063_t26 | LysoPC(20:0) | 0.09 | 0.02 | 0.00029209 | 0.03 | 2 | HMDB10390 |
| mz743.5767_t27.1 | NA | 0.09 | 0.02 | 0.00015487 | 0.02 | NA | NA |
| mz164.0739_t32.6 | S-(2-carboxypropyl)-Cysteamine | 0.09 | 0.02 | 8.41E-05 | 0.02 | 2 | HMDB02169 |
| mz88.0393_t70.5 | L-Aspartic acid | 0.09 | 0.02 | 9.77E-05 | 0.02 | 3; 2 | HMDB00191 |
| mz136.0427_t53.5 | Homocysteine | 0.10 | 0.02 | 2.00E-05 | 0.01 | 3 | HMDB00742 |
| mz551.4216_t23 | (3R3R6R9-cis)-Carotene-33-diol | 0.10 | 0.02 | 1.31E-05 | 0.01 | 3 | HMDB02198 |
| mz119.0171_t61.8 | Dihydro-4-mercapto-3(2H)-furanone | 0.11 | 0.02 | 6.88E-06 | 0.00 | 3 | HMDB39786 |
| mz150.0766_t62.1 | Methyladenine | 0.11 | 0.02 | 4.08E-06 | 0.00 | 2 | HMDB02099 |
| mz343.2855_t30.2 | NA | 0.11 | 0.02 | 5.61E-06 | 0.00 | NA | NA |
| mz180.0061_t53.8 | Homocysteine | 0.13 | 0.02 | 6.00E-08 | 0.00 | 3 | HMDB00742 |
